# Exposures to FD&C synthetic color additives from over-the-counter medications and vitamins in United States children and pregnant women

**DOI:** 10.1038/s41370-022-00418-9

**Published:** 2022-03-01

**Authors:** Ruwan Thilakaratne, Rosemary Castorina, Mayela Gillan, Dorothy Han, Teja Pattabhiraman, Anuroop Nirula, Mark D. Miller, Melanie Marty, Arlie Lehmkuhler, Alyson Mitchell, Asa Bradman

**Affiliations:** 1 Center for Environmental Research and Community Health (CERCH), School of Public Health, University of California at Berkeley, Berkeley, CA USA; 2 California Office of Environmental Health Hazard Assessment, Oakland, CA USA; 3 Department of Food Science and Technology, University of California at Davis, Davis, CA USA; 4 Department of Public Health, School of Social Sciences, Humanities, and Art, University of California at Merced, Merced, CA USA

**Keywords:** Human exposure, Artificial food colors, Hyperactivity, Children, Pregnant women, Acceptable daily intake

## Abstract

**Background:**

Food and Drug Administration (FDA) Food Drug and Cosmetic (FD&C) synthetic color additives (SCAs) have been associated with attentional and behavioral problems in children. Efforts to quantify exposure have focused on foods, while the contribution of medications and supplements remains unknown.

**Objective:**

To estimate exposures to SCAs in children (2–16 years) and pregnant women from intake of common over-the-counter (OTC) medications and vitamins.

**Methods:**

We estimated single-day exposure (mg/kg/day) to FD&C SCAs based on measurements of 25 different products and recommended dosages on product labels. Exposures were compared to SCA exposure estimates from food we previously developed and acceptable daily intakes (ADIs) established by FDA and the World Health Organization.

**Results:**

The highest exposure was found for FD&C Red No. 40 in a children’s cold/cough/allergy syrup. A child aged 12–16 years consuming the maximum daily dosage would have an exposure of 0.221 mg/kg/day, which is twice this age group’s typical exposure to this additive from food. No estimated exposures exceeded the ADIs.

**Significance:**

Some children’s OTC medications and vitamins may cause daily SCA exposures comparable to those from foods. OTC medications and vitamins should be considered in efforts to quantify population exposure to FD&C SCAs.

**Impact:**

Exposure to synthetic color additives (SCAs) from foods has been associated with behavioral problems in children. Exposures from over-the-counter (OTC) medications and vitamins remain unquantified despite widespread use. We estimated exposures in children and pregnant women for 25 different OTC medication and vitamin products sold in the United States. While exposures were below acceptable daily intakes (ADIs) established by the US Food and Drug Administration and the World Health Organization, some were comparable to typical daily exposures from foods. This work critically informs future SCA exposure assessments and provides valuable information for parents concerned about the health effects of SCAs.

## Introduction

Regulated synthetic color additives (SCAs) are artificial dyes that impart color in foods, drugs, and cosmetics. SCAs serve a variety of purposes, including enhancing the visual appeal of foods and facilitating the identification and differentiation of drugs. The Federal Food, Drug, and Cosmetic Act (FD&C) requires the United States Food and Drug Administration (FDA) to test the purity and composition of certain FD&C SCAs used in products sold in the United States through a process known as batch certification. Currently, nine SCAs require batch certification for use in foods and ingested medications sold in the United States. As two of these SCAs (Orange B and Citrus Red No. 2) are extremely limited in their use, the present study focuses on the other seven SCAs: FD&C Blue No. 1 (Brilliant Blue), FD&C Blue No. 2 (Indigo Carmine), FD&C Green No. 3 (Fast Green), FD&C Red No. 3 (Erythrosine), FD&C Red No. 40 (Allura Red), FD&C Yellow No. 5 (Tartrazine), and FD&C Yellow No. 6 (Sunset Yellow).

FD&C SCA consumption has raised public health concerns because of consistent links to adverse impacts on child behavior and activity [1]. Epidemiologic evidence for this has mounted over decades of research. A meta-analysis of 24 randomized controlled trials found statistically significant pooled associations between increased SCA consumption and attention-deficit/hyperactivity disorder (ADHD)-related symptoms in children [2]. Additionally, studies in which children diagnosed with ADHD were temporarily placed on diets eliminating SCA exposure showed an attenuation of ADHD symptoms [2]. A number of animal studies are consistent with these epidemiologic findings [3, 4]. Additionally, the FDA and the Joint Food and Agriculture Organization of the United Nations/World Health Organization Expert Committee on Food Additives (JECFA) have examined the impacts of SCAs on human health and established daily intake limits (acceptable daily intakes or ADIs) for each FD&C SCA based on toxicity studies in animals, though notably none of the ADIs are based on neurobehavioral endpoints [5–9].

Several studies have estimated population exposure to FD&C SCAs from food in the United States [1, 10–14]. However, exposures resulting from the use of pharmaceuticals have been largely unexplored. To our knowledge, only two studies have measured SCAs in drugs or vitamins [15, 16], and the resulting human exposures have not been quantified for children or pregnant women. Dietary supplements, such as vitamins, are typically consumed on a daily basis for long periods of time, resulting in chronic exposures to SCAs. This is concerning given that as much as one third of U.S. children take dietary supplements [17]. Furthermore, the dose regimens recommended on the product labels of over-the-counter (OTC) drugs can result in multiple intakes per day for multiple days or weeks, resulting in acute or sub-chronic exposures to SCAs, with surveys assessing OTC consumption in children suggesting their use is widespread [18, 19]. Thus, it is possible that these products could contribute significantly to some children’s daily SCA exposure and increase the overall population distribution of SCA exposure. Understanding the extent of exposure from vitamins and OTC medications can inform consumer decision-making and the design of future exposure assessment studies.

We previously reported concentrations of FD&C SCAs in a broad range of OTC medications and vitamins marketed to children and pregnant women [15]. In the present study, we aimed to estimate the SCA exposures (mg of SCA per kg of body weight per day, or mg/kg/day) that would result from using these products according to the recommended daily dosages on their labeling. Using standard reference body weights, we computed daily exposures for both children of multiple age ranges as well as pregnant women to account for prenatal exposure [20]. Estimates were compared to FDA and JECFA ADIs, as well as exposures from foods that we previously quantified [1]. This work was part of a larger health risk assessment of SCA exposure conducted by the California Office of Environmental Health Hazard Assessment [1].

## Methods

### Over-the-counter medications and vitamins

We collected a convenience sample of OTC medications and vitamins marketed to children and pregnant women. We reviewed major retailer websites, examined products on store shelves, and called retailers to identify products with at least one FD&C SCA listed on the ingredient label. Retailers included Target, CVS Pharmacy, Walmart, and RiteAid Pharmacy. To examine variability in SCA content across different batches of the same product, we required three distinct lot numbers of a given product for the product to be included in the analysis. Lot numbers are defined by expiration dates and manufacturing codes. Different lot numbers were obtained by visiting multiple retailers and sampling in several states, including California, Indiana, and Georgia. Several major categories of products were identified, typically with multiple brands per product category, including children’s pain reliever/fever reducer syrups, cold/cough/allergy syrups, gummy vitamins, pain reliever/fever reducer tablets, and allergy tablets, as well as prenatal vitamin tablets and softgels. In total, 25 OTC medication and vitamin products were identified.

The products were analyzed for the five FD&C SCAs (FD&C Red No. 40, FD&C Blue No. 1, FD&C Blue No. 2, FD&C Yellow No. 5, FD&C Yellow No. 6) found across their ingredient labels, by Dr. Alyson Mitchell’s laboratory at the University of California, Davis. The other two FD&C SCAs, FD&C Red No. 3 and FD&C Green No. 3, were not found on the ingredient labels of any of the products and therefore the products were not analyzed for these SCAs. SCA concentrations (mg of SCA per kg of product) were measured using laboratory methods that are described in detail elsewhere [15]. The measured concentrations have been published previously [21]. We adapted some of this data to present the averages and ranges of SCA concentrations measured across lot numbers of children’s pain reliever/fever reducer syrups and children’s cold/cough/allergy syrups in the Supplementary Information Tables S1 and S2, respectively. We present these tables as examples of the concentration data, and elected to display these product categories as they were found to have the highest concentrations of SCAs, and therefore the variability in concentrations across their lot numbers is the most consequential for exposure compared to other product categories.

### Exposure estimation

We first averaged concentrations for a given SCA across lot numbers for a given product (Tables S1 and S2). Then, SCA daily intake estimates (mg of SCA/day) were calculated by multiplying the average SCA concentration for syrups (mg/mL) or tablets, gummies, or softgels (mg/kg) by the volume or weight corresponding to a single age-specific recommended dose of the product, and then multiplying by the minimum or maximum number of daily recommended doses as defined by the product label. The result was divided by the age-specific median United States Environmental Protection Agency reference body weight value from the Exposure Factors Handbook [22] to derive SCA exposure (mg of SCA/kg of body weight/day). This exposure assessment approach has been used for OTC medications and vitamins [15, 16]. We calculated exposure estimates for the specific age categories provided on the product label of children’s OTC medications and vitamins, utilizing the age-specific dose information on product labels and matching age categories on product labels with age categories in the Exposure Factors Handbook as closely as possible. For prenatal vitamins, trimester-specific and overall average weights were used from the Exposure Factors Handbook. Formally, exposure estimates for syrups (1) and tablets, gummies, or softgels (abbreviated “tablets”) (2) were calculated using the following formulae:

1 $$\frac{{{{{{{\rm{color}}}}}}\,{{{{{\rm{additive}}}}}}\,({{{{{\rm{mg}}}}}})}}{{{{{{{\rm{body}}}}}}\,{{{{{\rm{weight}}}}}}\,\left( {{{{{{\rm{kg}}}}}}} \right) \cdot {{{{{\rm{day}}}}}}}} =	 \frac{{{{{{{\rm{color}}}}}}\,{{{{{\rm{additive}}}}}}\,({{{{{\rm{mg}}}}}})}}{{{{{{{\rm{syrup}}}}}}\,({{{{{\rm{mL}}}}}})}} * {{{{{\rm{dose}}}}}}_{{{{{{\rm{syrup}}}}}}}\left( {{{{{{\rm{mL}}}}}}} \right) \\ 	* \frac{{{{{{{\rm{doses}}}}}}}}{{{{{{{\rm{day}}}}}}}} * \frac{1}{{{{{{{\rm{body}}}}}}\,{{{{{\rm{weight}}}}}}\,({{{{{\rm{kg}}}}}})}}$$

2 $$\frac{{{{{{{\rm{color}}}}}}\,{{{{{\rm{additive}}}}}}\,({{{{{\rm{mg}}}}}})}}{{{{{{{\rm{body}}}}}}\,{{{{{\rm{weight}}}}}}\,\left( {{{{{{\rm{kg}}}}}}} \right) \cdot {{{{{\rm{day}}}}}}}} =	 \frac{{{{{{{\rm{color}}}}}}\,{{{{{\rm{additive}}}}}}\,({{{{{\rm{mg}}}}}})}}{{{{{{{\rm{tablet}}}}}}\,({{{{{\rm{kg}}}}}})}} * {{{{{\rm{dose}}}}}}_{{{{{{\rm{tablet}}}}}}}\left( {{{{{{\rm{kg}}}}}}} \right) \\ 	* \frac{{{{{{{\rm{doses}}}}}}}}{{{{{{{\rm{day}}}}}}}} * \frac{1}{{{{{{{\rm{body}}}}}}\,{{{{{\rm{weight}}}}}}\,({{{{{\rm{kg}}}}}})}}$$

where “dose” refers to a single, age-specific recommended OTC medication or vitamin dose listed on the product label, and “doses/day” refers to the number of those doses potentially consumed in a single day, namely the age-specific minimum or maximum number of doses listed on the product label.

### Comparisons to exposure estimates from food

To compare medication and food exposures, we calculated SCA exposures from OTC medications and vitamins as a percentage of SCA exposures from food. We limited this comparison to FD&C Red No. 40 because exposures to this SCA were consistently higher than for other SCAs among the products we studied. The lowest and highest FD&C Red No. 40 exposure estimates for a given product category were divided by the median two-day average daily exposure to FD&C Red No. 40 from foods for the corresponding child age group or pregnant women to obtain a range of the comparison percentage. The exposure estimates from foods were drawn from a nationally representative assessment of SCA exposure from foods we recently conducted, with methods described in detail elsewhere [1]. Briefly, the assessment linked SCA concentrations measured in a wide variety of foods sold by major US grocers [11] to two days of dietary recall data reported in the 2015–2016 National Health and Nutrition Examination Survey to estimate daily exposures.

Daily FD&C Red No. 40 exposures from foods for children and pregnant women are presented in Table S3. We utilized food-based estimates from a typical exposure scenario, which assumes consumers are exposed to the average SCA content of a given category of food, defined by averaging SCA concentrations over all products in that food category; the scenario is described further in a footnote under Table S3. In cases where an OTC medication or vitamin age range overlapped with multiple food-based exposure age ranges, we used the food-based exposure estimate from the age category that minimized (for the lower bound) or maximized (for the upper bound) the comparison percentage. For prenatal vitamins, the overall exposures for all pregnant women were used rather than trimester-specific exposures, as we were not able to derive trimester-specific food-based exposures.

### Maximum exposure estimates based on highest measured SCA concentration for a given product

We calculated maximum exposures by using the highest SCA concentration of the three lot numbers for a given product, in contrast to average exposures that use the average SCA concentration across lot numbers, to examine the impact of within-product manufacturing variability on exposures. We only performed this calculation for children’s products because we generally found lower exposures for pregnant women and using the maximum SCA concentrations for their products would be relatively inconsequential to the resulting exposures. Unless otherwise specified, references to exposure estimates in the “Results” section are for average exposures rather than maximum exposures.

### Interquartile range of exposure based on percentiles of reference body weights

For products that we found to cause higher exposures, namely pain reliever/fever reducer syrups and cold/cough/allergy syrups for children, we characterized the distribution of exposure within each age category using the 75th and 25th percentiles of body weight to compute the interquartile range (IQR) of exposure (25th and 75th percentiles of exposure, respectively), instead of the median body weight. IQRs of exposure were calculated using both the average SCA concentration across lot numbers and the maximum concentration.

### Comparisons to acceptable daily intakes

We compared estimated exposures from OTC medications and vitamins to US FDA and JECFA ADIs to identify any exceedances. The ADIs are presented in Table S4.

FD&C SCAs that are not mentioned under a given product category in the “Results” section were not identified on the ingredient labels for any of those products. All calculations for this study were performed in Microsoft Excel version 16.0.

## Results

### Children’s pain reliever/fever reducer syrups

Table 1 presents the FD&C Red No. 40 and FD&C Blue No. 1 exposure estimates (mg/kg/day) from five brands of children’s pain reliever/fever reducer syrups. The highest estimated exposures to these SCAs for children 2 to <11 years old were from Brand 2, grape-flavored syrup. The estimated FD&C Red No. 40 exposures for Brand 2 ranged from 0.029 to 0.032 mg/kg/day for 1 dose/day and 0.101 to 0.129 mg/kg/day for 4 doses/day. The estimated FD&C Blue No. 1 exposures for Brand 2 ranged from 0.005 to 0.006 mg/kg/day for 1 dose/day and 0.018 to 0.023 mg/kg/day for 4 doses/day. FD&C Red No. 40 exposure estimates from this product category ranged from 3 to 99% of those from foods [1] (Table S3).

**Table 1:** Children’s estimated exposures to FD&C Red No. 40 and FD&C Blue No. 1 synthetic color additives from recommended daily dosages of five brands of pain reliever/fever reducer syrups.

	FD&C Red No. 40 average exposure estimate (mg/kg/day)^a^		FD&C Blue No. 1 average exposure estimate (mg/kg/day)^a^	
	1 dose/day	4 doses/day	1 dose/day	4 doses/day
Brand 1, Berry				
2–3 years	0.004	0.017	ND	ND
4–5 years	0.005	0.019	ND	ND
6–8 years	0.004	0.015	ND	ND
9–10 years	0.005	0.018	ND	ND
Brand 2, Grape				
2–3 years	0.029	0.116	0.005	0.021
4–5 years	0.032	0.129	0.006	0.023
6–8 years	0.025	0.101	0.005	0.018
9–10 years	0.031	0.126	0.006	0.023
	**1 dose/day**	**5 doses/day**	**1 dose/day**	**5 doses/day**
Brand 3, Bubblegum				
2–3 years	0.009	0.044	ND	ND
4–5 years	0.010	0.049	ND	ND
6–8 years	0.008	0.039	ND	ND
9–10 years	0.010	0.048	ND	ND
Brand 4, Grape				
2–3 years	ND	ND	0.0001	0.0007
4–5 years	ND	ND	0.0002	0.0008
6–8 years	ND	ND	0.0001	0.0006
9–10 years	ND	ND	0.0002	0.0008
Brand 5, Bubblegum				
2–3 years	0.012	0.062	ND	ND
4–5 years	0.014	0.069	ND	ND
6–8 years	0.011	0.054	ND	ND
9–10 years	0.014	0.068	ND	ND

*FD&C* Food, Drug, and Cosmetic, *ND* non-detect, meaning the color additive is neither listed nor found in the product.

^a^Exposures are calculated as mg of color additive per kg of body weight per day (mg/kg/day). Body weights are age-specific median US Environmental Protection Agency reference body weights. Average exposure estimates are based on the average color additive concentration across three different lot numbers of the product.

### Children’s cold/cough/allergy syrups

Table 2 presents the FD&C Red No. 40 and FD&C Blue No. 1 exposure estimates from five brands of children’s cold/cough/allergy syrups. The highest estimated exposures to FD&C Red No. 40 and FD&C Blue No.1 for children 4 to 16 years old were from the Brand 5, grape-flavored syrup. The estimated FD&C Red No. 40 exposures from Brand 5 ranged from 0.028 to 0.037 mg/kg/day for 1 dose/day and 0.169 to 0.221 mg/kg/day for 6 doses/day. The estimated FD&C Blue No. 1 exposures from Brand 5 ranged from 0.004 to 0.005 mg/kg/day for 1 dose/day and 0.022 to 0.029 mg/kg/day for 6 doses/day. FD&C Red No. 40 exposure estimates from this product category ranged from 1 to 201% of those from foods (Table S3).

**Table 2:** Children’s estimated exposures to FD&C Red No. 40 and FD&C Blue No. 1 synthetic color additives from recommended daily dosages of five brands of cold/cough/allergy syrups.

	FD&C Red No. 40 average exposure estimate (mg/kg/day)^a^		FD&C Blue No. 1 average exposure estimate (mg/kg/day)^a^	
	1 dose/day	6 doses/day	1 dose/day	6 doses/day
Brand 1, Grape				
6–11 years	0.007	0.040	0.001	0.007
12–16 years	0.007	0.044	0.001	0.007
Brand 2, Cherry				
6–11 years	0.001	0.006	ND	ND
12–16 years	0.001	0.007	ND	ND
Brand 3, Grape				
6–<12 years	0.010	0.062	0.002	0.011
12–16 years	0.012	0.070	0.002	0.012
Brand 4, Very Berry				
6–<12 years	0.015	0.088	0.00004	0.0003
12–16 years	0.016	0.098	0.00005	0.0003
Brand 5, Grape				
4–6 years	0.028	0.169	0.004	0.022
6–<12 years	0.033	0.198	0.004	0.026
12–16 years	0.037	0.221	0.005	0.029

*FD&C* Food, Drug, and Cosmetic, *ND* non-detect, meaning the color additive is neither listed nor found in the product.

^a^Exposures are calculated as mg of color additive per kg of body weight per day (mg/kg/day). Body weights are age-specific median US Environmental Protection Agency reference body weights. Average exposure estimates are based on the average color additive concentration across three different lot numbers of the product.

### Children’s pain reliever/fever reducer tablets

Table 3 presents the FD&C Blue No. 1 and FD&C Blue No. 2 exposure estimates from three brands of children’s pain reliever/fever reducer tablets. The highest estimated exposure to FD&C Blue No. 1 was from Brand 1. The estimated FD&C Blue No. 1 exposure from Brand 1 for the minimum dosage ranged from 0.002 to 0.003 mg/kg/day and exposure for the maximum dosage ranged from 0.01 to 0.02 mg/kg/day for children 2–11 years old. Brand 2 was the only children’s pain reliever/fever reducer tablet product reported to contain FD&C Blue No. 2 on the ingredient label, but concentrations were below the detection limit.

**Table 3:** Children’s estimated exposures to FD&C Blue No. 1 and FD&C Blue No. 2 synthetic color additives from recommended daily dosages of three brands of pain reliever/fever reducer tablets.

Child’s age	FD&C Blue No. 1 average exposure estimate (mg/kg/day)^a^		FD&C Blue No. 2 average exposure estimate (mg/kg/day)^a^		FD&C Blue No. 1 average exposure estimate (mg/kg/day)^a^	
	Brand 1		Brand 2		Brand 3	
2–3 years	1 tablet/day (minimum)	0.003	–	NC	1 tablet/day (minimum)	0.0008
	5 tablets/day (maximum)	0.01	–	NC	4 tablets/day (maximum)	0.003
4–5 years	1.5 tablets/day (minimum)	0.003	–	NC	1.5 tablets/day (minimum)	0.0008
	7.5 tablets/day (maximum)	0.02	–	NC	6 tablets/day (maximum)	0.003
6–8 years	2 tablets/day (minimum)	0.002	2 tablets/day (minimum)	BDL	2 tablets/day (minimum)	0.001
	10 tablets/day (maximum)	0.01	8 tablets/day (maximum)	BDL	8 tablets/day (maximum)	0.003
9–10 years	2.5 tablets/day (minimum)	0.003	2.5 tablets/day (minimum)	BDL	2.5 tablets/day (minimum)	0.001
	12.5 tablets/day (maximum)	0.02	10 tablets/day (maximum)	BDL	10 tablets/day (maximum)	0.003
11 years	3 tablets/day (minimum)	0.002	3 tablets/day (minimum)	BDL	3 tablets/day (minimum)	0.0006
	15 tablets/day (maximum)	0.010	12 tablets/day (maximum)	BDL	12 tablets/day (maximum)	0.002

*FD&C* Food, Drug, and Cosmetic, *NC* not calculated as Brand 2 is not recommended for children under 6 years old, *BDL* below detection limit.

^a^Exposures are calculated as mg of color additive per kg of body weight per day (mg/kg/day). Body weights are age-specific median US Environmental Protection Agency reference body weights. Average exposure estimates are based on the average color additive concentration across three different lot numbers of the product.

### Children’s allergy tablets

Exposure estimates for FD&C Blue No. 1 and FD&C Blue No. 2 from four brands of children’s allergy tablets are presented in Table S5. The highest estimated exposure was 0.004 mg/kg/day of FD&C Blue No. 1 from the maximum dosage (12 tablets/day) for Brand 2 consumed by children 6–8 or 9–10 years old. FD&C Blue No. 2 was present on the ingredient label for the other three brands, but concentrations were below the detection limit.

### Children’s vitamin gummies

FD&C Red No. 40, FD&C Blue No. 1, FD&C Yellow No. 5, and FD&C Yellow No. 6 exposure estimates from three brands of children’s gummy vitamins are presented in Table 4. The highest exposure estimates were found for FD&C Red No. 40 from Brand 3 vitamins, ranging from 0.02 to 0.07 mg/kg/day for the minimum dosage of 1 gummy/day, and from 0.04 to 0.11 mg/kg/day for the maximum dosage of 2 gummies/day. FD&C Red. No. 40 exposure estimates from this product category ranged from 6 to 85% of typical exposures from foods (Table S3).

**Table 4:** Children’s estimated exposures to FD&C Red No. 40, FD&C Blue No. 1, FD&C Yellow No. 5, and FD&C Yellow No. 6 synthetic color additives from recommended daily dosages of three brands of gummy vitamins.

	FD&C Red No. 40 average exposure estimate (mg/kg/day)^a^		FD&C Blue No. 1 average exposure estimate (mg/kg/day)^a^		FD&C Yellow No. 5 average exposure estimate (mg/kg/day)^a^		FD&C Yellow No. 6 average exposure estimate (mg/kg/day)^a^	
	1 gummy /day	2 gummies /day	1 gummy /day	2 gummies /day	1 gummy /day	2 gummies /day	1 gummy /day	2 gummies /day
Brand 1 (Red, Orange and Purple)								
2–<3 years	0.04	NC	0.002	NC	ND	ND	ND	ND
3–<6 years	0.03	0.05	0.002	0.003	ND	ND	ND	ND
6–<11 years	0.02	0.03	0.001	0.002	ND	ND	ND	ND
11–<16 years	0.01	0.02	0.001	0.001	ND	ND	ND	ND
Brand 2 (Red, Orange and Purple)								
2–<3 years	0.03	NC	0.002	NC	ND	ND	0.01	NC
3–<6 years	0.02	0.04	0.001	0.002	ND	ND	0.007	0.01
6–<11 years	0.01	0.02	0.001	0.001	ND	ND	0.004	0.008
11–<16 years	0.007	0.01	0.001	0.001	ND	ND	0.003	0.005
Brand 3 (Red, Yellow and Green)								
2–<3 years	0.07	NC	0.004	NC	0.02	NC	ND	ND
3–<6 years	0.06	0.11	0.003	0.005	0.01	0.02	ND	ND
6–<11 years	0.03	0.06	0.002	0.003	0.01	0.01	ND	ND
11–<16 years	0.02	0.04	0.001	0.002	0.004	0.008	ND	ND

*FD&C* Food, Drug, and Cosmetic, *ND* non-detect, meaning the color additive is neither listed nor found in the product, *NC* not calculated as 2 gummies per day is not recommended for children under 3 years of age.

^a^Exposures are calculated as mg of color additive per kg of body weight per day (mg/kg/day). Body weights are age-specific median US Environmental Protection Agency reference body weights. Average exposure estimates are based on the average color additive concentration across three different lot numbers of the product.

### Prenatal vitamin tablets

Pregnant women’s SCA exposure estimates from prenatal vitamin tablets are presented in Table 5. Estimated exposures were generally lower than exposures from children’s OTC medications or vitamins. The highest estimate was for FD&C Red No. 40 from Brand 1 vitamins, ranging from 0.0025 to 0.0028 mg/kg/day for trimester-specific exposures and averaged 0.0027 mg/kg/day for all pregnant women consuming one tablet per day. FD&C Red No. 40 exposure estimates from this product category ranged from 4 to 7% of typical exposure for pregnant women from foods (Table S3).

**Table 5:** Pregnant women’s estimated exposures to FD&C Blue No. 2, FD&C Red No. 40, and FD&C Yellow No. 6 synthetic color additives from recommended daily dosages of four brands of prenatal vitamin tablets.

	FD&C Blue No. 2 average exposure (mg/kg/day)^a^	FD&C Red No. 40 average exposure (mg/kg/day)^a^	FD&C Yellow No. 6 average exposure (mg/kg/day)^a^
	1 tablet/day	1 tablet/day	1 tablet/day
Brand 1			
1st trimester	ND	0.0026	3.6E−06
2nd trimester	ND	0.0028	3.7E−06
3rd trimester	ND	0.0025	3.4E−06
Average for all pregnant women	ND	0.0027	3.6E−06
Brand 2			
1st trimester	BDL	0.0013	0.0002
2nd trimester	BDL	0.0013	0.0002
3rd trimester	BDL	0.0012	0.0002
Average for all pregnant women	BDL	0.0013	0.0002
Brand 3			
1st trimester	ND	0.0021	0.0003
2nd trimester	ND	0.0022	0.0003
3rd trimester	ND	0.0020	0.0004
Average for all pregnant women	ND	0.0022	0.0004
Brand 4			
1st trimester	8.9E−06	0.0018	0.0004
2nd trimester	9.2E−06	0.0019	0.0004
3rd trimester	8.4E−06	0.0017	0.0004
All pregnant women	9.0E−06	0.0019	0.0004

*FD&C* Food, Drug, and Cosmetic, *ND* non-detect, meaning the color additive is neither listed nor found in the product, *BDL* below detection limit.

^a^Exposures are calculated as mg of color additive per kg of body weight per day (mg/kg/day). Body weights are trimester-specific and overall median US Environmental Protection Agency reference body weights. Average exposure estimates are based on the average color additive concentration across three different lot numbers of the product.

### Prenatal vitamin softgels

Exposure estimates for FD&C Blue No. 1 and FD&C Red No. 40 from one brand of prenatal vitamin softgels are presented in Table 6. Similar to prenatal vitamin tablets, SCA exposure estimates from prenatal vitamin softgels were lower than those from children’s OTC medications or vitamins. Exposures to FD&C Red No. 40 from prenatal vitamin softgels were greater than exposures to FD&C Blue No. 1. Trimester-specific estimated exposure to FD&C Red No. 40 for 1 softgel/day ranged from 0.0043 to 0.0048 mg/kg/day and averaged 0.0046 mg/kg/day for all pregnant women. FD&C Red No. 40 exposure estimates from this brand of prenatal vitamin softgel were 15% of typical exposure for pregnant women from foods (Table S3).

**Table 6:** Pregnant women’s estimated exposures to FD&C Blue No. 1 and FD&C Red No. 40 synthetic color additives from the recommended daily dosage of one brand of prenatal vitamin softgel.

	FD&C Blue No.1 average exposure (mg/kg/day)^a^	FD&C Red No. 40 average exposure (mg/kg/day)^a^
	1 softgel/day	1 softgel/day
Brand 1		
1st trimester	0.0006	0.0046
2nd trimester	0.0006	0.0048
3rd trimester	0.0005	0.0043
All pregnant women	0.0006	0.0046

*FD&C* Food, Drug, and Cosmetic.

^a^Exposures are calculated as mg of color additive per kg of body weight per day (mg/kg/day). Body weights are trimester-specific and overall median US Environmental Protection Agency reference body weights. Average exposure estimates are based on the average color additive concentration across three different lot numbers of the product.

### Maximum exposure estimates

Exposure estimates based on the maximum SCA concentration across lot numbers of a given children’s product were very similar to estimates based on the average of the three measurements within each lot (Tables S5–S9). The highest estimated exposure was to FD&C Red No. 40 in Brand 5 grape-flavored cold/cough/allergy syrup consumed by children 12–16 years old: the estimated exposure was 0.232 mg/kg/day compared to 0.221 mg/kg/day when using the average SCA concentration (Table S7).

### Interquartile range exposure estimates

Estimates of the IQR (25th and 75th percentiles) of exposure for children’s syrups, calculated using the 75th and 25th percentiles of body weight for a given age category, respectively, were generally similar to estimates based on the median body weight (Tables S10 and S11). The highest estimated exposure was to FD&C Red No. 40 in Brand 5 grape-flavored cold/cough/allergy syrup consumed by children 12–16 years old. The 75th percentile exposure estimate (25th percentile body weight) based on the product’s average FD&C Red No. 40 SCA concentration was 0.279 mg/kg/day (Table S11), compared to 0.221 mg/kg/day when using the median body weight (Table S7).

### Comparison of synthetic color additive exposure to acceptable daily intakes

No exposure estimates exceeded the US FDA or JECFA ADIs.

## Discussion

We estimated single-day exposures to FD&C SCAs associated with intake of manufacturer-recommended dosages of a broad range of 25 OTC medications and vitamins used by children and pregnant women in the United States. In the sampled products, FD&C Red No. 40, Blue No. 1, Blue No. 2, Yellow No. 5, and Yellow No. 6 were identified on ingredient labels, and were detected in laboratory measurements in most cases. Generally, estimated exposures were higher for children than for pregnant women. Consumption of children’s pain reliever/fever reducer syrups and cold/cough/allergy syrups resulted in the highest exposures, followed by children’s gummy vitamins, children’s pain reliever/fever reducer tablets, children’s allergy tablets, prenatal vitamin softgels, and prenatal vitamin tablets. Exposures were generally greater for FD&C Red No. 40 than other SCAs. None of the exposure estimates exceeded established FDA and JECFA ADIs, though some exposure estimates for FD&C Red No. 40 from children’s cold/cough/allergy syrups exceeded estimates of typical exposure to this SCA from foods. For children’s products, variability in SCA concentrations across batches of the same product and variability in reference body weights within age categories did not strongly impact estimated exposures.

To our knowledge, the present work represents the first effort to systematically characterize the distribution of exposures to FD&C SCAs from these commonly used and widely available medications and vitamins. The only other related work we are aware of is a study by Šuleková et al. [16], in which six different vitamin products sold in Europe were sampled and analyzed for their color additive content [16]. Among the color additives examined relevant to the US were FD&C Yellow No. 5 and FD&C Yellow No. 6, and exposures were estimated for an adult weighing 70 kg. Reported estimates were far below the JECFA and US FDA ADIs (Table S4). This work did not address vitamins designed for children, the population thought to be susceptible to possible neurobehavioral impacts of these compounds. The use of vitamins generates daily exposures, adding to chronic exposures from regular intake of foods high in SCA content, such as fruit drinks and icings [1, 11]. We found that among the products we sampled, vitamin-based SCA exposures were low relative to the ADIs, though for children’s cold/cough/allergy syrups, some exposure estimates exceeded typical exposure from foods.

It is likely that the consumption of OTC medications for acute illness would be of short duration, such as a few days or weeks. Regular consumption of some medications for persistent conditions, such as allergy medications or non-steroidal anti-inflammatory drugs, however, would result in sub-chronic or chronic exposures. While we did not aim to enumerate actual OTC medication and vitamin consumption patterns in a population, previous work suggests the use of these products is very common in US children: in a sample of US children <12 years of age, more than half were reported to have used at least one medication (prescription or non-prescription) over the seven days preceding the interview [18, 19]. Among the most commonly consumed medications were cough/cold medications and vitamins, and OTC medications were used much more frequently than prescription medications.

While none of the estimated exposures exceeded US FDA and JECFA ADIs, these limits are based on chronic toxicity studies with non-neurobehavioral endpoints, such as measures of liver toxicity and thyroid hormone dysfunction [1]. Impacts on behavior have been observed in animal toxicology studies at exposure levels below the no observed adverse effect levels used to derive US FDA and JECFA ADIs, including for SCAs we found in OTC medications and vitamins, such as FD&C Red No. 40 [3] and FD&C Blue No. 1 [4]. Exposures from OTC medications and vitamins would become more significant in relation to the ADIs if the ADIs were revised using these studies.

The present study has several limitations. Our convenience sample was unable to represent the full range of OTC medication and vitamin products on the market that contain SCAs, such as products sold in local or regional markets and in states not encompassed by our sampling frame. However, our selection spans 25 different products across three different states, covering major retailers and brand names that likely constitute a significant share of the market for these products. Additionally, there was some incongruency between product dosing age ranges and reference body weight age ranges, which would result in under- and over-estimating exposures. However, these incongruencies are unlikely to impact comparisons to food-based SCA exposures or ADIs, as they never exceeded two years of age, and we found that varying body weight within age categories did not strongly influence estimated exposures.

OTC medications and vitamins contribute to children’s total daily SCA exposure, and for some populations and types of products, exposures to FD&C Red No. 40 may be similar in magnitude to those from foods. Parents concerned about the health effects of SCAs and parents of children with known SCA sensitivities should be made aware of their prevalence in these commonly used products, and of the availability of naturally colored or color-free alternatives for many products. OTC medications and vitamins should be considered in future population-level studies assessing exposures to FD&C SCAs. Particularly, estimates of acute and sub-chronic exposure may be significantly affected by these products. Furthermore, a more comprehensive and systematic survey of OTC medications and vitamins can better characterize the full spectrum of exposure from currently marketed products, better represent within-product variability in SCA content, and may identify FD&C Green No. 3 or FD&C Red No. 3, SCAs that were not found in the products we examined. The SCA content of prescription drugs may warrant investigation, as those used to treat chronic conditions, such as psychiatric disorders, may be taken on a near-daily basis for years [23]. Furthermore, we did not examine another group of SCAs, D&C (drug and cosmetic) SCAs, which are approved for use in drugs and cosmetics only, but not foods. While these were not the focus of our work, the potential health effects and population exposure levels of these compounds are not well understood, suggesting an additional avenue for research on children’s exposure to SCAs.

In conclusion, manufacturer-recommended consumption of OTC medications and vitamins alone are unlikely to result in exceedances of established ADIs, though single-day exposures to FD&C Red No. 40 may be comparable to single-day exposures to this SCA from foods. The findings of this study are informative for parents with concerns about the health effects of SCAs and suggest estimates of population-level exposure to SCAs would be improved by accounting for the contributions of OTC medications and vitamins.

### Supplementary Information

Supplementary Information
