# Supplementary material for: Exposures to FD&C synthetic color additives from over-the-counter medications and vitamins in United States children and pregnant women
Source: J Expo Sci Environ Epidemiol. 2022 Mar 1;33(5):787–93. doi: 10.1038/s41370-022-00418-9 (PMC10541320; doi:10.1038/s41370-022-00418-9)
Supplement: Supplementary file 1 — Supplementary Information [file 41370_2022_418_MOESM1_ESM.docx]

Supplementary Information

**Exposures to FD&C synthetic color additives from over-the-counter medications and vitamins in United States children and pregnant women**

Ruwan Thilakaratne^1^, Rosemary Castorina^1,*^, Dorothy Han^1^, Teja Pattabhiraman^1^, Anuroop Nirula^1^, Mark D. Miller^2^, Melanie Marty^2^, Arlie Lehmkuhler^3^, Alyson Mitchell^3^, Asa Bradman^1,4^

**Authors’ affiliation**

^1^Center for Environmental Research and Community Health (CERCH), School of Public Health, University of California at Berkeley, Berkeley, CA, USA.

^2^California Office of Environmental Health Hazard Assessment, Oakland, CA, USA.

^3^Department of Food Science & Technology, University of California at Davis, Davis, CA, USA.

^4^Department of Public Health, School of Social Sciences, Humanities, and Art, University of California at Merced, Merced, CA, USA.

***Corresponding author:**

Rosemary Castorina, PhD, MPH

Center for Environmental Research and Community Health (CERCH)

School of Public Health

University of California, Berkeley

1995 University Avenue, Suite 265

Berkeley, CA  94704

[rcastori@berkeley.edu](mailto:rcastori@berkeley.edu)

**16 pages, 11 tables**

Tables

[Table S1. Summary of FD&C synthetic color additive concentrations measured in five brands of children's pain reliever/fever reducer syrup over-the-counter medicines. 3](#_Toc93948418)

[Table S2. Summary of FD&C synthetic color additive concentrations measured in five brands of children's cold/cough/allergy syrup over-the-counter medicines. 4](#_Toc93948419)

[Table S3. Single-day and two-day average FD&C Red No. 40 exposure (mg/kg/day) from foods among United States pregnant women and children under typical- and high-exposure scenarios. 5](#_Toc93948420)

[Table S4. FD&C synthetic color additive acceptable daily intakes (ADIs) established by the United States Food and Drug Administration (US FDA) and Joint Food and Agriculture Organization of the United Nations/World Health Organization Expert Committee on Food Additives (JECFA). 6](#_Toc93948421)

[Table S5. Children’s estimated average and maximum exposures to FD&C Blue No. 1 and FD&C Blue No. 2 (mg/kg/day) synthetic color additives from recommended daily dosages of four brands of allergy tablets. 7](#_Toc93948422)

[Table S6. Children’s estimated average and maximum exposures to FD&C Red No. 40 and FD&C Blue No. 1 synthetic color additives from recommended daily dosages of five brands of pain reliever/fever reducer syrups. 8](#_Toc93948423)

[Table S7. Children’s estimated average and maximum exposures to FD&C Red No. 40 and FD&C Blue No. 1 synthetic color additives from recommended daily dosages of five brands of cold/cough/allergy syrups. 9](#_Toc93948424)

[Table S8. Children’s estimated average and maximum exposures to FD&C Blue No. 1 and FD&C Blue No. 2 synthetic color additives from recommended daily dosages of three brands of pain reliever/fever reducer tablets. 10](#_Toc93948425)

[Table S9. Children’s estimated average and maximum exposures to FD&C Red No.40, FD&C Blue No. 1, FD&C Yellow No. 5, and FD&C Yellow No. 6 synthetic color additives from recommended daily dosages of three brands of gummy vitamins. 11](#_Toc93948426)

[Table S10. Children’s estimated interquartile range (25^th^ and 75^th^ percentiles) exposures to FD&C Red No. 40 and FD&C Blue No. 1 synthetic color additives from recommended daily dosages of five brands of pain relievers/fever reducer syrups, using average and maximum synthetic color additive concentration estimates. 12](#_Toc93948427)

[Table S11. Children’s estimated interquartile range (25^th^ and 75^th^ percentiles) exposures to FD&C Red No. 40 and FD&C Blue No. 1 synthetic color additives from recommended daily dosages of five brands of cold/cough/allergy syrups, using average and maximum synthetic color additive concentration estimates. 15](#_Toc93948428)

# Table S1. Summary of FD&C synthetic color additive concentrations measured in five brands of children's pain reliever/fever reducer syrup over-the-counter medicines.

| Brand | FD&C Red No. 40 concentration (mg/mL)^a^ | | FD&C Blue No. 1 concentration (mg/mL)^a^ | |
| --- | --- | --- | --- | --- |
|  | Average | Range | Average | Range |
| Brand 1 | 0.012 | 0.011-0.012 | ND | ND |
| Brand 2 | 0.08 | 0.077-0.085 | 0.014 | 0.014-0.016 |
| Brand 3 | 0.025 | 0.022-0.027 | ND | ND |
| Brand 4 | ND | ND | 0.0004 | 0.0002-0.0006 |
| Brand 5 | 0.034 | 0.032-0.037 | ND | ND |

FD&C: Food, Drug, & Cosmetic; ND: non-detect, meaning the color additive was neither listed nor found in the product

Note: Table includes measurements from three different lot numbers of each brand of syrup. Lot numbers are defined by expiration dates and manufacturing codes.

^a^mg of synthetic color additive per mL of syrup

Adapted from: Lehmkuhler AL, Miller MD, Bradman A, Castorina R, Mitchell AE. Dataset of certified food dye levels in over the counter medicines and vitamins intended for consumption by children and pregnant women. Data Brief. 2020 Oct 1;32:106073.

# Table S2. Summary of FD&C synthetic color additive concentrations measured in five brands of children's cold/cough/allergy syrup over-the-counter medicines.

| Brand | FD&C Red No. 40 concentration (mg/mL)^a^ | | FD&C Blue No. 1 concentration (mg/mL)^a^ | |
| --- | --- | --- | --- | --- |
|  | Average | Range | Average | Range |
| Brand 1 | 0.042 | 0.039-0.045 | 0.007 | 0.006-0.008 |
| Brand 2 | 0.006 | 0.006-0.007 | ND | ND |
| Brand 3 | 0.033 | 0.032-0.035 | 0.006 | 0.0056-0.0065 |
| Brand 4 | 0.093 | 0.081-0.114 | 0.0003 | 0.0001-0.0005 |
| Brand 5 | 0.105 | 0.099-0.110 | 0.014 | 0.013-0.015 |

FD&C: Food, Drug, & Cosmetic; ND: non-detect, meaning the color additive was neither listed nor found in the product

Note: Table includes measurements from three different lot numbers of each brand of syrup. Lot numbers are defined by expiration dates and manufacturing codes.

^a^mg of color additive per mL of syrup

Adapted from: Lehmkuhler AL, Miller MD, Bradman A, Castorina R, Mitchell AE. Dataset of certified food dye levels in over the counter medicines and vitamins intended for consumption by children and pregnant women. Data Brief. 2020 Oct 1;32:106073.

# Table S3. Single-day and two-day average FD&C Red No. 40 exposure (mg/kg/day) from foods among United States pregnant women and children under typical- and high-exposure scenarios.

| FD&C Red No. 40 |  |  | Typical-exposure scenario^d^ | | | High-exposure scenario^e^ | | |
| --- | --- | --- | --- | --- | --- | --- | --- | --- |
|  | Total n*^b^* | n^c^ | Mean | Median | 95th% | Mean | Median | 95th% |
| Pregnant women |  |  |  |  |  |  |  |  |
| Day 1 | 48 | 44 | 0.14 | 0.04 | 0.53 | 0.26 | 0.07 | 1.38 |
| Day 2 | 31 | 27 | 0.08 | 0.01 | 0.31 | 0.24 | 0.03 | 1.72 |
| 2-Day average*^a^* | 42 | 39 | 0.09 | 0.03 | 0.52 | 0.21 | 0.06 | 0.69 |
| Children (2-<5 years) |  |  |  |  |  |  |  |  |
| Day 1 | 388 | 366 | 0.30 | 0.16 | 0.91 | 0.66 | 0.23 | 3.28 |
| Day 2 | 300 | 265 | 0.30 | 0.18 | 0.92 | 0.73 | 0.32 | 3.02 |
| 2-Day average*^a^* | 363 | 352 | 0.23 | 0.13 | 0.75 | 0.52 | 0.25 | 2.04 |
| Children (5-<9 years) |  |  |  |  |  |  |  |  |
| Day 1 | 569 | 550 | 0.30 | 0.21 | 0.91 | 0.71 | 0.39 | 2.51 |
| Day 2 | 397 | 378 | 0.26 | 0.17 | 0.79 | 0.73 | 0.27 | 2.97 |
| 2-Day average*^a^* | 501 | 491 | 0.23 | 0.17 | 0.73 | 0.60 | 0.32 | 2.13 |
| Children (9-<16) years) |  |  |  |  |  |  |  |  |
| Day 1 | 908 | 860 | 0.20 | 0.14 | 0.63 | 0.52 | 0.25 | 2.05 |
| Day 2 | 660 | 622 | 0.20 | 0.13 | 0.68 | 0.56 | 0.23 | 2.72 |
| 2-Day average*^a^* | 843 | 822 | 0.16 | 0.11 | 0.51 | 0.44 | 0.23 | 1.63 |

FD&C: Food, Drug, and Cosmetic; SCA: synthetic color additive; NHANES: National Health and Nutrition Examination Survey (2015-2016)

*^a^*The 2-Day average estimates include individuals who completed both the Day 1 and Day 2 NHANES food consumption questionnaires.

*^b^*Total n=number of SCA eaters, i.e. individuals who ate at least one food containing any of the seven FD&C SCAs.

*^c^*n=number of individuals who consumed at least one food containing FD&C Red No. 40; means, medians, and 95^th^ percentiles are calculated based on these individuals.

^d^The typical-exposure scenario was calculated as follows: 1) for those foods whose SCA content was measured in triplicate, the average of the 3 measurements for each SCA was used, and 2) in cases where a single NHANES food code represented multiple foods, the average concentration of each SCA across all foods associated with that food code was assigned to that food code.

^e^The high-exposure scenario estimate was calculated as follows: 1) for those foods whose SCA content was measured in triplicate, the highest of the 3 measurements for each SCA was used; and 2) in cases where a single NHANES food code represented multiple foods, the maximum concentration of each SCA found across the foods associated with that food code was assigned to that food code.

Adapted from: OEHHA (Office on Environmental Health Hazard Assessment). Final Report. Environmental health risk assessment of artificial food colors for women of childbearing age, pregnant women and children [Internet]. 2020 Aug [cited 2021 May 20]. Available from: <https://oehha.ca.gov/risk-assessment/public-comment-period-general-info/announcement-release-public-review-draft-health>

# Table S4. FD&C synthetic color additive acceptable daily intakes (ADIs) established by the United States Food and Drug Administration (US FDA) and Joint Food and Agriculture Organization of the United Nations/World Health Organization Expert Committee on Food Additives (JECFA).

| **FD&C synthetic color additive** | **US FDA**  **(mg/kg/day)** | **JECFA**  **(mg/kg/day)** |
| --- | --- | --- |
| Blue No. 1 | 12.0 | 0-6 |
| Blue No. 2 | 2.5 | 0-5 |
| Green No. 3 | 2.5 | 0-25 |
| Red No. 3 | 2.5 | 0-0.1 |
| Red No. 40 | 7.0 | 0-7 |
| Yellow No. 5 | 5.0 | 0-10 |
| Yellow No. 6 | 3.75 | 0-4 |

FD&C: Food, Drug, and Cosmetic

Note: JECFA presents their ADIs as a range from 0 to a positive value.

# Table S5. Children’s estimated average and maximum exposures to FD&C Blue No. 1 and FD&C Blue No. 2 (mg/kg/day) synthetic color additives from recommended daily dosages of four brands of allergy tablets.

| Child's age | Dosage | FD&C Blue No. 2 Average (Max) Exposure Estimate (mg/kg/day)^a^ | | FD&C Blue No. 1  Average (Max) Exposure  Estimate (mg/kg/day) ^a^ | | FD&C Blue No. 2 Average (Max) Exposure Estimate (mg/kg/day) ^a^ | | FD&C Blue No. 2 Average (Max) Exposure Estimate (mg/kg/day) ^a^ | |
| --- | --- | --- | --- | --- | --- | --- | --- | --- | --- |
|  |  | Brand 1 | | Brand 2 | | Brand 3 | | Brand 4 | |
| 2-3 years | Minimum | 1 tablet/day | BDL | - | NC | 1 tablet/day | BDL | 1 tablet/day | BDL |
|  | Maximum | 1 tablet/day | BDL | - | NC | 1 tablet/day | BDL | 1 tablet/day | BDL |
| 4-5 years | Minimum | 1 tablet/day | BDL | - | NC | 1 tablet/day | BDL | 1 tablet/day | BDL |
|  | Maximum | 1 tablet/day | BDL | - | NC | 1 tablet/day | BDL | 1 tablet/day | BDL |
| 6-8 years | Minimum | 2 tablets/day | BDL | 1-2 tablets/day | 0.0004 -0.0007  (0.0006-0.001) | 2 tablets/day | BDL | 2 tablets/day | BDL |
|  | Maximum | 2 tablets/day | BDL | 12 tablets/day | 0.004  (0.008) | 2 tablets/day | BDL | 2 tablets/day | BDL |
| 9-10 years | Minimum | 2 tablets/day | BDL | 1-2 tablets/day | 0.0004-0.0007  (0.0006-0.001) | 2 tablets/day | BDL | 2 tablets/day | BDL |
|  | Maximum | 2 tablets/day | BDL | 12 tablets/day | 0.004  (0.008) | 2 tablets/day | BDL | 2 tablets/day | BDL |
| 11 years | Minimum | 2 tablets/day | BDL | 1-2 tablets/day | 0.0002-0.0004  (0.0004-0.0007) | 2 tablets/day | BDL | 2 tablets/day | BDL |
|  | Maximum | 2 tablets/day | BDL | 12 tablets/day | 0.003  (0.004) | 2 tablets/day | BDL | 2 tablets/day | BDL |

FD&C: Food, Drug, and Cosmetic; BDL: below detection limit; NC: not calculated, as Brand 2 allergy tablets are not recommended for children less than 6 years of age.

^a^ Exposures are calculated as mg of color additive per kg of body weight per day (mg/kg/day). Body weights are age-specific median US Environmental Protection Agency reference body weights. Average exposure estimates are based on the average color additive concentration across three different lot numbers of the product, whereas maximum exposure estimates are based on the maximum concentration among the lot numbers.

# Table S6. Children’s estimated average and maximum exposures to FD&C Red No. 40 and FD&C Blue No. 1 synthetic color additives from recommended daily dosages of five brands of pain reliever/fever reducer syrups.

|  | FD&C Red No. 40  Average (Max) Exposure Estimate (mg/kg/day)^a^ | | FD&C Blue No. 1  Average (Max) Exposure Estimate (mg/kg/day)^a^ | |
| --- | --- | --- | --- | --- |
|  | 1 dose/day | 4 doses/day | 1 dose/day | 4 doses/day |
| Brand 1, Berry |  |  |  |  |
| 2-3 years | 0.004 (0.004) | 0.017 (0.017) | ND | ND |
| 4-5 years | 0.005 (0.005) | 0.019 (0.019) | ND | ND |
| 6-8 years | 0.004 (0.004) | 0.015 (0.015) | ND | ND |
| 9-10 years | 0.005 (0.005) | 0.018 (0.019) | ND | ND |
| Brand 2, Grape |  |  |  |  |
| 2-3 years | 0.029 (0.031) | 0.116 (0.123) | 0.005 (0.006) | 0.021 (0.023) |
| 4-5 years | 0.032 (0.034) | 0.129 (0.137) | 0.006 (0.006) | 0.023 (0.026) |
| 6-8 years | 0.025 (0.027) | 0.101 (0.107) | 0.005 (0.005) | 0.018 (0.020) |
| 9-10 years | 0.031 (0.033) | 0.126 (0.134) | 0.006 (0.006) | 0.023 (0.025) |
|  |  |  |  |  |
|  | 1 dose/day | 5 doses/day | 1 dose/day | 5 doses/day |
| Brand 3, Bubblegum |  |  |  |  |
| 2-3 years | 0.009 (0.010) | 0.044 (0.049) | ND | ND |
| 4-5 years | 0.010 (0.011) | 0.049 (0.054) | ND | ND |
| 6-8 years | 0.008 (0.008) | 0.039 (0.042) | ND | ND |
| 9-10 years | 0.010 (0.011) | 0.048 (0.053) | ND | ND |
| Brand 4, Grape |  |  |  |  |
| 2-3 years | ND | ND | 0.0001 (0.002) | 0.0007 (0.0011) |
| 4-5 years | ND | ND | 0.0002 (0.002) | 0.0008 (0.0012) |
| 6-8 years | ND | ND | 0.0001 (0.002) | 0.0006 (0.0009) |
| 9-10 years | ND | ND | 0.0002 (0.002) | 0.0008 (0.0012) |
| Brand 5, Bubblegum |  |  |  |  |
| 2-3 years | 0.012 (0.013) | 0.062 (0.067) | ND | ND |
| 4-5 years | 0.014 (0.015) | 0.069 (0.075) | ND | ND |
| 6-8 years | 0.011 (0.012) | 0.054 (0.058) | ND | ND |
| 9-10 years | 0.014 (0.015) | 0.068 (0.073) | ND | ND |

FD&C: Food, Drug, and Cosmetic; ND: non-detect, meaning the color additive is neither listed nor found in the product

^a^ Exposures are calculated as mg of color additive per kg of body weight per day (mg/kg/day). Body weights are age-specific median US Environmental Protection Agency reference body weights. Average exposure estimates are based on the average color additive concentration across three different lot numbers of the product, whereas maximum exposure estimates are based on the maximum concentration among the lot numbers.

# Table S7. Children’s estimated average and maximum exposures to FD&C Red No. 40 and FD&C Blue No. 1 synthetic color additives from recommended daily dosages of five brands of cold/cough/allergy syrups.

|  | FD&C Red No. 40  Average (Max) Exposure Estimate (mg/kg/day)^a^ | | FD&C Blue No. 1  Average (Max) Exposure Estimate (mg/kg/day)^a^ | |
| --- | --- | --- | --- | --- |
|  | 1 dose/day | 6 doses/day | 1 dose/day | 6 doses/day |
| Brand 1, Grape |  |  |  |  |
| 6-11 years | 0.007 (0.007) | 0.040 (0.042) | 0.001 (0.001) | 0.007 (0.008) |
| 12-16 years | 0.007 (0.008) | 0.044 (0.048) | 0.001 (0.001) | 0.007 (0.008) |
| Brand 2, Cherry |  |  |  |  |
| 6-11 years | 0.001 (0.001) | 0.006 (0.007) | ND | ND |
| 12-16 years | 0.001 (0.001) | 0.007 (0.007) | ND | ND |
| Brand 3, Grape |  |  |  |  |
| 6-<12 years | 0.010 (0.011) | 0.062 (0.066) | 0.002 (0.002) | 0.011 (0.012) |
| 12-16 years | 0.012 (0.012) | 0.070 (0.074) | 0.002 (0.002) | 0.012 (0.014) |
| Brand 4, Very Berry |  |  |  |  |
| 6-<12 years | 0.015 (0.018) | 0.088 (0.108) | 0.00004 (0.00008) | 0.0003 (0.0005) |
| 12-16 years | 0.016 (0.020) | 0.098 (0.120) | 0.00005 (0.00009) | 0.0003 (0.0005) |
| Brand 5, Grape |  |  |  |  |
| 4-6 years | 0.028 (0.030) | 0.169 (0.177) | 0.004 (0.004) | 0.022 (0.024) |
| 6-<12 years | 0.033 (0.035) | 0.198 (0.208) | 0.004 (0.005) | 0.026 (0.028) |
| 12-16 years | 0.037 (0.039) | 0.221 (0.232) | 0.005 (0.005) | 0.029 (0.032) |

FD&C: Food, Drug, and Cosmetic; ND: non-detect, meaning the color additive is neither listed nor found in the product

^a^ Exposures are calculated as mg of color additive per kg of body weight per day (mg/kg/day). Body weights are age-specific median US Environmental Protection Agency reference body weights. Average exposure estimates are based on the average color additive concentration across three different lot numbers of the product, whereas maximum exposure estimates are based on the maximum concentration among the lot numbers.

# Table S8. Children’s estimated average and maximum exposures to FD&C Blue No. 1 and FD&C Blue No. 2 synthetic color additives from recommended daily dosages of three brands of pain reliever/fever reducer tablets.

| Child's age | Dosage | FD&C Blue No. 1 Average (Max) Exposure Estimate (mg/kg/day)^a^ | | FD&C Blue No. 2 Average (Max) Exposure Estimate (mg/kg/day)^a^ | | FD&C Blue No. 1 Average (Max) Exposure Estimate (mg/kg/day)^a^ | |
| --- | --- | --- | --- | --- | --- | --- | --- |
|  |  | Brand 1 | | Brand 2 | | Brand 3 | |
| 2-3 years | Minimum dosage | 1 tablet/day | 0.003 (0.003) | - | NC | 1 tablet/day | 0.0008 (0.0009) |
|  | Maximum recommended dosage | 5 tablets/day | 0.01 (0.01) | - | NC | 4 tablets/day | 0.003  (0.004) |
| 4-5 years | Minimum dosage | 1.5 tablets/day | 0.003 (0.003) | - | NC | 1.5 tablets/day | 0.0008  (0.001) |
|  | Maximum recommended dosage | 7.5 tablets/day | 0.02  (0.02) | - | NC | 6 tablets/day | 0.003  (0.004) |
| 6-8 years | Minimum dosage | 2 tablets/day | 0.002  (0.003) | 2 tablets/day | BDL | 2 tablets/day | 0.001  (0.001) |
|  | Maximum recommended dosage | 10 tablets/day | 0.01  (0.01) | 8 tablets/day | BDL | 8 tablets/day | 0.003  (0.003) |
| 9-10 years | Minimum dosage | 2.5 tablets/day | 0.003  (0.003) | 2.5 tablets/day | BDL | 2.5 tablets/day | 0.001  (0.001) |
|  | Maximum recommended dosage | 12.5 tablets/day | 0.02  (0.02) | 10 tablets/day | BDL | 10 tablets/day | 0.003  (0.004) |
| 11 years | Minimum dosage | 3 tablets/day | 0.002  (0.002) | 3 tablets/day | BDL | 3 tablets/day | 0.0006  (0.0006) |
|  | Maximum recommended dosage | 15 tablets/day | 0.010  (0.011) | 12 tablets/day | BDL | 12 tablets/day | 0.002  (0.003) |

FD&C: Food, Drug, and Cosmetic; NC: not calculated, because Brand 2 is not recommended for children under 6 years old; BDL: below detection limit

^a^ Exposures are calculated as mg of color additive per kg of body weight per day (mg/kg/day). Body weights are age-specific median US Environmental Protection Agency reference body weights. Average exposure estimates are based on the average color additive concentration across three different lot numbers of the product, whereas maximum exposure estimates are based on the maximum concentration among the lot numbers.

# Table S9. Children’s estimated average and maximum exposures to FD&C Red No.40, FD&C Blue No. 1, FD&C Yellow No. 5, and FD&C Yellow No. 6 synthetic color additives from recommended daily dosages of three brands of gummy vitamins.

|  | FD&C Red No. 40  Average (Max) Exposure Estimate (mg/kg/day)^a^ | | FD&C Blue No. 1  Average (Max) Exposure Estimate (mg/kg/day)^a^ | | FD&C Yellow No. 5  Average (Max) Exposure Estimate (mg/kg/day)^a^ | | FD&C Yellow No. 6  Average (Max) Exposure Estimate (mg/kg/day)^a^ | |
| --- | --- | --- | --- | --- | --- | --- | --- | --- |
|  | 1 gummy /day | 2 gummies /day | 1 gummy /day | 2 gummies /day | 1 gummy /day | 2 gummies /day | 1 gummy /day | 2 gummies /day |
| Brand 1 (Red, Orange & Purple) | | |  |  |  |  |  |  |
| 2-<3 years | 0.04 (0.05) | NC | 0.002 (0.003) | NC | ND | ND | ND | ND |
| 3-<6 years | 0.03 (0.04) | 0.05 (0.08) | 0.002 (0.002) | 0.003 (0.004) | ND | ND | ND | ND |
| 6 -<11 years | 0.02 (0.03) | 0.03 (0.05) | 0.001 (0.001) | 0.002 (0.002) | ND | ND | ND | ND |
| 11-<16 years | 0.01 (0.02) | 0.02 (0.03) | 0.001 (0.001) | 0.001 (0.001) | ND | ND | ND | ND |
| Brand 2 (Red, Orange & Purple) | | |  |  |  |  |  |  |
| 2-<3 years | 0.03 (0.04) | NC | 0.002 (0.002) | NC | ND | ND | 0.01 (0.02) | NC |
| 3-<6 years | 0.02 (0.03) | 0.04 (0.06) | 0.001 (0.002) | 0.002 (0.003) | ND | ND | 0.007 (0.01) | 0.01 (0.02) |
| 6 -<11 years | 0.01 (0.02) | 0.02 (0.04) | 0.001 (0.001) | 0.001 (0.002) | ND | ND | 0.004 (0.007) | 0.008 (0.01) |
| 11-<16 years | 0.007 (0.01) | 0.01 (0.02) | 0.001 (0.001) | 0.001 (0.001) | ND | ND | 0.003 (0.004) | 0.005 (0.007) |
| Brand 3 (Red, Yellow & Green) | | |  |  |  |  |  |  |
| 2-<3 years | 0.07 (0.086) | NC | 0.004 (0.005) | NC | 0.02 (0.02) | NC | ND | ND |
| 3-<6 years | 0.06 (0.07) | 0.11 (0.13) | 0.003 (0.004) | 0.005 (0.007) | 0.01 (0.02) | 0.02 (0.03) | ND | ND |
| 6 -<11 years | 0.03 (0.04) | 0.06 (0.08) | 0.002 (0.002) | 0.003 (0.004) | 0.01 (0.01) | 0.01 (0.02) | ND | ND |
| 11-<16 years | 0.02 (0.02) | 0.04 (0.04) | 0.001 (0.001) | 0.002 (0.002) | 0.004 (0.005) | 0.008 (0.01) | ND | ND |

FD&C: Food, Drug, and Cosmetic; ND: not detected, meaning the color additive is neither listed nor found in the product; NC: not calculated, because 2 gummy vitamins per day is not recommended for children under 3 years of age

^a^ Exposures are calculated as mg of color additive per kg of body weight per day (mg/kg/day). Body weights are age-specific median US Environmental Protection Agency reference body weights. Average exposure estimates are based on the average color additive concentration across three different lot numbers of the product, whereas maximum exposure estimates are based on the maximum concentration among the lot numbers.

| Table S10. Children’s estimated interquartile range (25^th^ and 75^th^ percentiles) exposures to FD&C Red No. 40 and FD&C Blue No. 1 synthetic color additives from recommended daily dosages of five brands of pain relievers/fever reducer syrups, using average and maximum synthetic color additive concentration estimates. | | | | |
| --- | --- | --- | --- | --- |
|  | FD&C Red No. 40 | | FD&C Blue No. 1 | |
|  | IQR_average_ (IQR_max_) Exposure Estimate (mg/kg/day)^a^ | | IQR_average_ (IQR_max_) Exposure Estimate (mg/kg/day)^a^ | |
|  | 1 dose/day | 4 doses/day | 1 dose/day | 4 doses/day |
| Brand 1, Berry |  | |  | |
| 2-3 years | 0.004-0.005 | 0.016-0.019 | ND | ND |
|  | (0.004-0.005) | (0.016-0.019) |  |  |
| 4-5 years | 0.004-0.005 | 0.017-0.022 | ND | ND |
|  | (0.004-0.006) | (0.018-0.023) |  |  |
| 6-8 years | 0.003-0.005 | 0.013-0.019 | ND | ND |
|  | (0.003-0.005) | (0.013-0.020) |  |  |
| 9-10 years | 0.004-0.006 | 0.016-0.024 | ND | ND |
|  | (0.004-0.006) | (0.016-0.025) |  |  |
| Brand 2, Grape |  | |  | |
| 2-3 years | 0.027-0.032 | 0.107-0.129 | 0.005-0.006 | 0.019-0.023 |
|  | (0.029-0.034) | (0.114-0.137) | (0.005-0.006) | (0.021-0.026) |
| 4-5 years | 0.030-0.038 | 0.118-0.152 | 0.005-0.007 | 0.021-0.028 |
|  | (0.031-0.040) | (0.126-0.161) | (0.006-0.008) | (0.024-0.030) |
| 6-8 years | 0.022-0.033 | 0.087-0.131 | 0.004-0.006 | 0.016-0.024 |
|  | (0.023-0.035) | (0.092-0.139) | (0.004-0.007) | (0.017-0.026) |
| 9-10 years | 0.027-0.041 | 0.109-0.164 | 0.005-0.007 | 0.020-0.030 |
|  | (0.029-0.044) | (0.115-0.174) | (0.005-0.008) | (0.022-0.033) |
| Brand 3, Bubblegum | | | | |
| 2-3 years | 0.008-0.010 | 0.041-0.049 | ND | ND |
|  | (0.009-0.011) | (0.045-0.054) |  |  |
| 4-5 years | 0.009-0.012 | 0.045-0.058 | ND | ND |
|  | (0.010-0.013) | (0.050-0.064) |  |  |
| 6-8 years | 0.007-0.010 | 0.033-0.050 | ND | ND |
|  | (0.007-0.011) | (0.037-0.055) |  |  |
| 9-10 years | 0.008-0.013 | 0.042-0.063 | ND | ND |
|  | (0.009-0.014) | (0.046-0.069) |  |  |
| Brand 4, Grape |  | |  | |
| 2-3 years | ND | ND | 0.0001-0.0002 | 0.0007-0.0008 |
|  |  |  | (0.0002-0.0002) | (0.0010-0.0012) |
| 4-5 years | ND | ND | 0.0001-0.0002 | 0.0007-0.0008 |
|  |  |  | (0.0002-0.0002) | (0.0010-0.0012) |
| 6-8 years | ND | ND | 0.0001-0.0002 | 0.0007-0.0009 |
|  |  |  | (0.0002-0.0003) | (0.0011-0.0014) |
| 9-10 years | ND | ND | 0.0001-0.0002 | 0.0005-0.0008 |
|  |  |  | (0.0002-0.0002) | (0.0008-0.0012) |
| Brand 5, Bubblegum | | |  | |
| 2-3 years | 0.012-0.014 | 0.058-0.069 | ND | ND |
|  | (0.012-0.015) | (0.062-0.075) |  |  |
| 4-5 years | 0.013-0.016 | 0.064-0.082 | ND | ND |
|  | (0.014-0.018) | (0.068-0.088) |  |  |
| 6-8 years | 0.009-0.014 | 0.047-0.070 | ND | ND |
|  | (0.010-0.015) | (0.050-0.076) |  |  |
| 9-10 years | 0.012-0.018 | 0.058-0.088 | ND | ND |
|  | (0.013-0.019) | (0.063-0.095) |  |  |
| FD&C: Food, Drug, and Cosmetic; IQR: interquartile range; ND: non-detect, meaning the color additive was neither listed nor found in the product; SCA: synthetic color additive | | | | |
| ^a^ ­25^th^ and 75^th^ percentiles (IQR) of exposure were calculated using the 75^th^ and 25^th^ percentiles, respectively, of child age-specific reference body weights from the United States Environmental Protection Agency. IQR_average_ is the IQR based on the average SCA concentration (averaged across three different lot numbers of the product), whereas IQR_max_ is the IQR based on the maximum SCA concentration (the maximum concentration among the three lot numbers).   \| Table S11. Children’s estimated interquartile range (25^th^ and 75^th^ percentiles) exposures to FD&C Red No. 40 and FD&C Blue No. 1 synthetic color additives from recommended daily dosages of five brands of cold/cough/allergy syrups, using average and maximum synthetic color additive concentration estimates. \| \| \| \| \| \| --- \| --- \| --- \| --- \| --- \| \|  \| FD&C Red No. 40 \| \| FD&C Blue No. 1 \| \| \| IQR_average_ (IQR_max_) Exposure Estimate (mg/kg/day)^a^ \| \| IQR_average_ (IQR_max_) Exposure Estimate (mg/kg/day)^a^ \| \| \| 1 dose/day \| 6 doses/day \| 1 dose/day \| 6 doses/day \| \| Brand 1, Grape \|  \| \|  \| \| \| 6-11 years \| 0.006-0.009 \| 0.034-0.052 \| 0.001-0.001 \| 0.006-0.009 \| \| (0.006-0.009) \| (0.037-0.055) \| (0.001-0.002) \| (0.007-0.010) \| \| 12-16 years \| 0.006-0.009 \| 0.039-0.056 \| 0.001-0.002 \| 0.006-0.009 \| \| (0.007-0.010) \| (0.042-0.060) \| (0.001-0.003) \| (0.007-0.011) \| \| Brand 2, Cherry \|  \| \|  \| \| \| 6-11 years \| 0.001-0.0009 \| 0.005-0.008 \| ND \| ND \| \| (0.001-0.001) \| (0.006-0.009) \| \| 12-16 years \| 0.001-0.001 \| 0.006-0.008 \| ND \| ND \| \| (0.001-0.002) \| (0.006-0.009) \| \| Brand 3, Grape \|  \| \|  \| \| \| 6-<12 years \| 0.009-0.014 \| 0.054-0.081 \| 0.002-0.002 \| 0.010-0.015 \| \| (0.010-0.014) \| (0.057-0.086) \| (0.002-0.003) \| (0.011-0.016) \| \| 12-16 years \| 0.010-0.015 \| 0.061-0.088 \| 0.002-0.003 \| 0.011-0.016 \| \| (0.011-0.016) \| (0.065-0.093) \| (0.002-0.003) \| (0.012-0.017) \| \| Brand 4, Very Berry \| \| \| \| \| \| 6-<12 years \| 0.009-0.014 \| 0.054-0.081 \| 0.00004-0.00005 \| 0.0002-0.0003 \| \| (0.010-0.014) \| (0.057-0.086) \| (0.00007-0.0001) \| (0.0004-0.0006) \| \| 12-16 years \| 0.010-0.015 \| 0.061-0.088 \| 0.00004-0.00006 \| 0.0002-0.0004 \| \| (0.011-0.016) \| (0.065-0.093) \| (0.00008-0.0001) \| (0.0005-0.0007) \| \| Brand 5, Grape \|  \| \|  \| \| \| 4-6 years \| 0.026-0.033 \| 0.155-0.199 \| 0.003-0.004 \| 0.020-0.026 \| \| (0.027-0.035) \| (0.163-0.209) \| (0.004-0.005) \| (0.022-0.028) \| \| 6-<12 years \| 0.028-0.043 \| 0.171-0.258 \| 0.004-0.006 \| 0.023-0.034 \| \| (0.030-0.045) \| (0.179-0.270) \| (0.004-0.006) \| (0.024-0.037) \| \| 12-16 years \| 0.032-0.047 \| 0.193-0.279 \| 0.004-0.006 \| 0.026-0.037 \| \| (0.034-0.049) \| (0.203-0.293) \| (0.005-0.007) \| (0.028-0.040) \| \| FD&C: Food, Drug, and Cosmetic; IQR: interquartile range; ND: non-detect, meaning the color additive was neither listed nor found in the product; SCA: synthetic color additive \| \| \| \| \| \| ^a^ ­25^th^ and 75^th^ percentiles (IQR) of exposure were calculated using the 75^th^ and 25^th^ percentiles, respectively, of child age-specific reference body weights from the United States Environmental Protection Agency. IQR_average_ is the IQR based on the average SCA concentration (averaged across three different lot numbers of the product), whereas IQR_max_ is the IQR based on the maximum SCA concentration (the maximum concentration among the three lot numbers). \| \| \| \| \| | | | | |
